# Construction of Aggregation-Induced Emission Molecule–MnO_2_ Composite Nanoprobe and Its Application in Alkaline Phosphatase Detection

**DOI:** 10.3390/nano13142138

**Published:** 2023-07-23

**Authors:** Yanyun Cui, Jun Zhao, Huidan Li

**Affiliations:** College of Chemistry and Materials Engineering, Beijing Technology and Business University, Beijing 100048, China; zhaojun@st.btbu.edu.cn (J.Z.); lihuidan@st.btbu.edu.cn (H.L.)

**Keywords:** aggregation-induced emission, fluorescence sensor, alkaline phosphatase, SiO_2_ nanospheres, MnO_2_ nanosheet

## Abstract

Alkaline phosphatase (ALP) is among the most studied enzymes by far, playing an important role in the metabolism of organisms and the regulation of protein activity. Herein, a label-free composite nanoprobe is constructed by combining inorganic nanomaterials and aggregation-induced emission (AIE) molecule to achieve highly sensitive and selective detection of ALP. Negatively charged 9,10-bis [2-(6-sulfonatopropoxyl) naphthylethenyl] anthracene (BSNVA) molecule is synthesized, which has the AIE performance and can be assembled on the surface of amino–SiO_2_ nanoparticles through electrostatic interaction for fluorescence enhancement. MnO_2_ nanosheets are rich in negative charges, enabling them to be wrapped on the surface of the amino–SiO_2_ nanosphere to shield the positive charge on its surface, making it impossible for BSNVA to accumulate on the surface and then weakening the bio-fluorescence of the system. Furthermore, with catalyzed substrates induced by ALP, generating ascorbic acid and the redox reaction between ascorbic acid and MnO_2_, the nanoprobe helps in realizing the high-sensitivity detection of ALP with a detection limit of 0.38 mU/mL. The proposed strategy requires no complex cleaning and modification processes and can overcome the quenching effect caused by the aggregation of traditional organic dyes, proving to be a simple, low-cost and “turn-on” fluorescent detection method for ALP.

## 1. Introduction

Alkaline phosphatase (ALP) is one of the most commonly assayed biological enzymes, which can catalyze the hydrolysis process of a variety of substrates, such as proteins, nucleic acids and monophosphates, and plays a pivotal role in the metabolism of organisms and the regulation of protein activity [1]. In addition, ALP is an important biomarker closely related to the diagnosis of many diseases. Normally, the serum ALP concentration of normal adults is 40–190 U/L, while those of children and pregnant women can reach 500 U/L [2]. A variety of diseases have been found to be associated with abnormal ALP activity, such as bone cancer, prostate cancer, liver disease, breast cancer, nasopharyngeal carcinoma, colorectal cancer and pancreatic cancer [3,4,5,6,7,8,9,10]. Therefore, the detection of ALP activities is of great significance in clinical diagnosis and disease treatment. In recent years, a large number of ALP detection sensor systems have been reported, including colorimetric sensors, electrochemical sensors, surface-enhanced Raman scattering sensors and fluorescence sensors [11,12,13,14,15]. Although these methods can be applied in the detection of ALP, they all have some inherent defects. For instance, colorimetric sensors are developed based on gold nanoparticles (hence, they are susceptible to salts and other materials in the environment); electrochemical sensors require complex cleaning and modifications of electrodes; and surface-enhanced Raman scattering sensors require specialized and sophisticated instruments. In contrast, fluorescence-sensing systems have drawn extensive attention from researchers due to their advantages such as simple operation, less sample consumption and real-time detection. At present, inorganic quantum dots, metal–organic frameworks, coordination polymers and traditional organic dyes have been successfully used in the development of ALP fluorescent sensors [16,17,18,19,20,21]. However, the current design of sensing systems still has many defects, such as the complex synthesis and modification process of fluorophores, the high toxicity of inorganic quantum dots and the poor solubility of traditional organic dyes in aqueous solution. Moreover, most highly concentrated and gathered fluorescent molecules are prone to the ACQ effect, which limits the practical application of ALP sensors.

In recent years, many fluorescence enhancement techniques have been used in the construction of high sensitivity sensors. For example, the plasmonically coupled sensors constructed by Amirjani et al. based on gold and silver nanoparticles demonstrate four orders of magnitude greater sensitivity towards ferricyanide [22]. A Zn (II)-based metal–organic framework synthesized by Yao et al. can sensitively and selectively sense acetylacetone via a fluorescence enhancement effect [23]. Luo et al. developed an efficient fluorescence-enhanced probe for the detection of cyanide ion (CN^-^) based on tetraphenyl coordination copper iodide complex [24]. Aggregation-induced emission (AIE) molecules have drawn wide public and research attention due to their unique advantages over traditional dyes, such as large Stokes shift, lower toxicity and sound stability [25]. More importantly, they are non-emissive in the dispersed state, but emit strong fluorescence after aggregation, which reduces the background interference and increases the signal-to-noise ratio, thus improving the method’s sensitivity. These advantages allow them to be widely used in biosensing and bioimaging [26,27]. Thanks to the above advantages, the Han research group synthesized TPE derivatives modified by glucosamine hydrochloride [28]. In the presence of amphiphilic compounds, TPE derivatives can aggregate and glow. In the presence of ALP, the phosphate of the compound is hydrolyzed, which leads to aggregate decomposition. However, the modification process of this specific functional group is complex and the scope of application is narrow. Moreover, the quenching fluorescence sensor can easily be disturbed by the background and other substances in detection. Therefore, the development of an enhanced fluorescence sensor based on the induced hydrolysis performance of ALP is of great significance for the high-sensitivity detection of ALP.

Silica nanoparticles are commonly used as inorganic nanomaterials. Due to their advantages of easy synthesis, easy modification, sound stability and biocompatibility, they are often used as carriers for various chemical and biological species [29,30]. In recent years, manganese dioxide (MnO_2_) nanosheets, a type of two-dimensional inorganic material, have attracted people’s attention and been widely used in various fields, including biological analysis and cell imaging [31]. MnO_2_ nanosheets have the advantages of low cost, simple preparation process, sound biocompatibility and large specific surface area [31,32,33]. In addition, the surface of the MnO_2_ nanosheet has a large amount of negative charge; hence, it can absorb materials with positive charge. Furthermore, MnO_2_ nanosheets are very sensitive to reducing substances and can easily be reduced and decomposed, making them the ideal materials for building sensitive biosensor systems [32,34]. 

With all the above information, an AIE molecule 9,10-bis [2-(6-proloxylsulfonate)-naphthalenevinyl] anthracenesulfonate sodium salt (BSNVA) was combined with inorganic nanomaterials to construct a label-free and enhanced fluorescent sensor for ALP detection in this work. The surface of amino–SiO_2_ nanoparticles was positively charged, enabling negatively charged BSNVA to accumulate on the surface and enhance fluorescence. A MnO_2_ nanosheet could be wrapped on the surface of the amino–SiO_2_ nanoparticles, so as to shield the positive charge on the surface of the silicon ball and weaken the fluorescence of the system. By introducing suitable substrates and ALP, the detection of ALP was realized based on the interaction between the hydrolysate induced by ALP and the MnO_2_ nanosheet. Compared with the traditional fluorescence detection methods, this method overcomes various disadvantages such as the aggregation caused quenching of traditional organic dye and poor light stability. It is a label-free and enhanced fluorescence sensing method, with high sensitivity and selectivity for the detection of ALP.

## 2. Materials and Methods

### 2.1. Chemicals and Materials

1,3-Propanesultone, (3-Aminopropyl) triethoxysilane (APTES, 98%) and sodium ethoxide were obtained from Alfa Aesar (Shanghai, China). Tetraethyl orthosilicate (TEOS), hexadecyltrimethylammonium bromide (CTAB), reduced N-ethylmaleimide (NEM) and 2-phospho-L-ascorbic acid (AAP) were purchased from Sigma-Aldrich (Shanghai, China). MnCl_2_·4H_2_O purchased from Beijing Chemical Works (Beijing, China). L-Ascorbic acid (AA) was purchased from InnoChem (Beijing, China). Tetramethylammonium hydroxide solution was purchased from Aladdin Bio-Chem Technology Co., Ltd. (Shanghai, China). Ethanol (99.5%, SuperDry) was obtained from J&K Scientific Ltd. (Beijing, China). All reagents were purchased from suppliers without further purification. Deionized water (18.2 MΩ cm) was purified using Milli-Q water purification.

### 2.2. Instrumentation

UV–vis spectra were characterized by a UV-2450 spectrometer (Shimadzu, Kyoto, Japan). Fluorescence spectra were measured using an RF-6000 (Shimadzu, Japan). The excitation wavelength was 410 nm and both the excitation and emission slits had a width of 5 nm. Transmission electron microscopy (TEM) images were measured on a JEOL 2010 transmission electron microscope operated at an accelerating voltage of 200 kV. The sample used for TEM characterization was prepared by dropping a suspension on a carbon-coated copper grid and drying at room temperature. Scanning electron microscope (SEM) images were measured on an S-8010 (Hitachi, Kyoto, Japan). The ^1^H NMR spectra were measured on a JEOL 400 MHz spectrometer with DMSO (dimethyl sulfoxide) as the solvent. The zeta potential measurements were recorded with a Nano-ZS Zetasizer ZEN3600 (Malvern Instruments Ltd., Malvern, UK).

### 2.3. Preparation of MnO_2_–SiO_2_ NPs

In a typical experiment, the preparation of amino-functionalized SiO_2_ nanospheres (SiO_2_–NH_2_ NSs) was carried out through the Stöber process [35]. First, under the condition of stirring, NH_3_·H_2_O (0.1 M, 200 μL) was added to CTAB (0.08 M, 50 mL) and the mixture was evenly mixed. Then, 0.08 mL of 20% TEOS methanol solution was rapidly added to the mixture and stirred at room temperature for 20 h. The mixture was centrifuged, washed and dried to obtain white SiO_2_ nanospheres. Next, 120 mg of the SiO_2_ nanospheres was added to the ethanol and stirred to fully dissolve it. Then, 30 μL of APTES was added to the solution and vigorously stirred for 10 h. The above solution was centrifuged and washed with ethanol three times to remove excess APTES, and the precipitate was dried to obtain SiO_2_–NH_2_ NSs.

MnO_2_ nanosheets were synthesized following the method described in the literature [36]. MnCl_2_·4H_2_O (0.5945 g) was dissolved in water (10 mL) and then 20 mL of the mixture of tetramethylammonium hydroxide solution (12.35 mL) and H_2_O_2_ (2 mL, 30 wt. %) was added. The mixture solution immediately turned brown and was stirred at room temperature for 12 h. The precipitate was collected by centrifugation, then washed with methanol and water and dried to obtain massive MnO_2_. The bulk MnO_2_ solid (0.0100 g) was dispersed in 20 mL distilled water and the MnO_2_ solution was centrifuged at 2000 rpm/min for 10 min after 12 h ultrasonic treatment. The MnO_2_ nanosheets were removed from the supernatant and stored at 4 °C for future use. 

Then, 0.0050 g of SiO_2_–NH_2_ NSs and 0.0050 g of MnO_2_ nanosheets were dissolved in 10 mL of distilled water and dispersed by ultrasound. The dispersed SiO_2_–NH_2_ NSs solution (40 μL) and nanosheets solution (20 μL) were mixed and incubated at room temperature for 30 min to form the complex. The mixed solution was centrifuged and washed three times to remove the unbound and unstable MnO_2_ nanosheets. The precipitates were dispersed into the same volume of water to prepare MnO_2_-SiO_2_ NPs solution.

### 2.4. Synthesis of 9,10-Bis [2-(6-sulfonatopropoxyl)naphthylethenyl]anthracene

BSNVA was synthesized following the methods of our previous work [37]. The synthesis route is shown in Figure S1. The reaction took place in a nitrogen atmosphere, and 9,10-bis [2-(6-hydroxy)naphthyl-ethenyl]anthracene (0.515 g, 1 mmol) was taken as the reactant and dissolved in anhydrous ethanol (20 mL). Then, the anhydrous ethanol solution of sodium ethanolate (20 mL, 2.3 mmol) was added to the mixture dropwise. The solution was stirred until it turned orange-red, then 1,3-propanesultone (200 μL) was added. The mixed solution was stirred until the product was separated out. The filtered precipitate was then washed with ethanol and acetone to form 9,10-bis [2-(6-sulfonatopropoxyl)naphthylethenyl] anthracene (BSNVA), verified via ^1^H NMR (400 MHz, DMSO) δ 8.45 (dd, *J* = 8.0, 4.5 Hz, 4H), 8.20 (d, *J* = 16.5 Hz, 2H), 8.17–8.03 (m, 4H), 7.82 (dd, *J* = 8.7, 5.0 Hz, 4H), 7.59 (dd, *J* = 6.9, 3.2 Hz, 4H), 7.19–7.13 (m, 4H), 7.12 (d, *J* = 16.5 Hz, 2H), 7.07 (d, *J* = 16.5 Hz, 2H), 4.08 (t, *J* = 6.5 Hz, 4H), 2.64 (t, *J* = 7.4 Hz, 4H), 2.03–1.97 (m, 4H).

### 2.5. The Detection of ALP with MnO_2_-SiO_2_–AIE Nanocomposite System

First, 5 μL of different concentrations of ALP (with a final concentration at 0, 1, 5, 10, 20, 40, 80, 120, 160, 200, 300, 400 or 500 mU/mL) and 10 μL of AAP solution (60 mM) was mixed with 60 μL of MnO_2_–SiO_2_ complex, then incubated under 37 °C for 20 min. After adding 25 μL of BSNVA (20 μg/mL), the mixture was then incubated at room temperature for 15 min for adequate reaction. The fluorescence intensity of the system was recorded by a fluorescence spectrometer. The excitation wavelength was 410 nm.

### 2.6. Detection of ALP in Human Serum Sample

The serum samples were diluted 100-fold with PBS buffer (10 mM, pH = 7.4) and incubated with NEM (0.2 mM) for 20 min at 37 °C. Then, different concentrations of ALP were added to the above solution for recovery tests. The measurement method and reaction conditions were the same as those for ALP detection above.

## 3. Results and Discussion

**Scheme 1:**
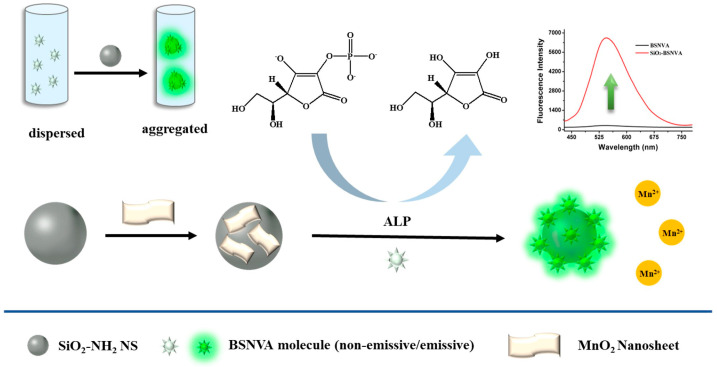
Schematic illustration of the AIE–MnO_2_ composite nanosystem for alkaline phosphatase detection.

The construction of the AIE–MnO_2_ composite nanosystem is illustrated in Scheme 1. The prepared BSNVA molecules were water-soluble and hardly emitted any light when dispersed in water. Due to the presence of a large number of amino groups on the surface of the SiO_2_–NH_2_ NSs, they are positively charged and can bind negatively charged BSNVA through electrostatic interaction, so that BSNVA can accumulate on the surface of the SiO_2_–NH_2_ NSs and emit strong green fluorescence. The MnO_2_ nanosheet is an inorganic two-dimensional nanomaterial with excellent biocompatibility that can be reduced to Mn^2+^ by AA and then decomposed; hence, it is widely used in the construction of biosensors. In addition, ALP catalyzes the process of dephosphorylation by removing the phosphate groups from the substrate to form hydroxyl groups. Based on the above principles, a MnO_2_ nanosheet is introduced into the sensing system. A MnO_2_ nanosheet has a negative charge and can be wrapped on the surface of SiO_2_–NH_2_ NSs through electrostatic adsorption, so as to shield the positive charge on the surface and avoid binding with BSNVA, resulting in no luminescence in the system. With AAP and ALP coexisting, ALP can catalyze the former to generate AA, thereby inducing the decomposition of the MnO_2_ nanosheet. Meanwhile, SiO_2_–NH_2_ NSs are released, BSNVA accumulates on the surface of SiO_2_–NH_2_ NSs and fluorescence is enhanced. The high sensitivity and specificity of ALP can be achieved following the dynamics of fluorescence intensity of the system.

### 3.1. Preparation and Characterization of Nanoparticles

**Figure 1:**
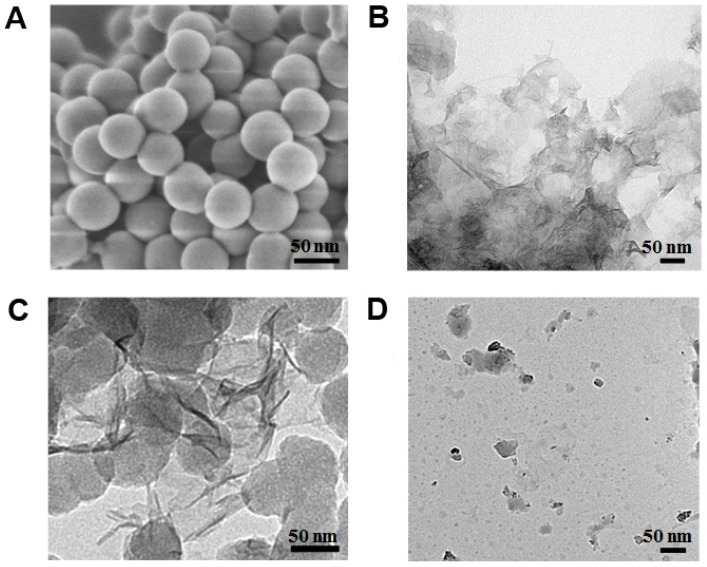
(**A**) SEM image of SiO_2_–NH_2_ NSs, (**B**) TEM image of MnO_2_ nanosheets, (**C**) TEM image of MnO_2_ nanosheet-modified SiO_2_ assemblies and (**D**) TEM image of MnO_2_ nanosheets in the presence of AA.

SiO_2_–NH_2_ NSs and MnO_2_ nanosheets were synthesized and their sizes and morphology were observed by scanning electron microscopy (SEM) and transmission electron microscopy (TEM). As shown in Figure 1A, the synthesized SiO_2_–NH_2_ NSs were spherical and had good dispersion in aqueous solution, with a mean diameter of 57.9 nm. The morphology of the MnO_2_ nanosheet is shown in Figure 1B: the nanosheet was a two-dimensional lamellar structure with some folds. SiO_2_–NH_2_ NSs can be adsorbed on the surface of a MnO_2_ nanosheet through electrostatic interaction to form a MnO_2_-SiO_2_ complex; zeta potential measurements revealed that the potential changed (Figure S2). TEM images showed that the MnO_2_ nanosheet had been successfully coated on the surface of SiO_2_–NH_2_ NSs (Figure 1C). In addition, it can be clearly seen from Figure 1D that the MnO_2_ nanosheet was decomposed in the presence of AA, which is consistent with our expected results.

### 3.2. Synthesis and Characterization of Anthracene Derivatives

The water-soluble molecule BSNVA was synthesized, with a large conjugated structure cored by 9,10-divinylanthracene, and its hydrogen on vinyl replaced by naphthalene (Figure S1). Its structure was confirmed by ^1^H NMR (Figure S3). In order to explain its luminescence mechanism, the structure of BSNVA was optimized using the Gaussian 09 procedure and B3LYP method. As shown in Figure S4, the optimal structure of BSNVA presents a distorted conformation. When BSNVA is in the dissolved state, the vibration of 9,10-divinylanthracene and the rotation of the peripheral naphthalene can dissipate the energy of the excited state. In addition, the highly distorted conformation makes it easier for the peripheral aromatic ring of BSNVA to rotate in the aqueous solution, thus reducing the fluorescence yield. However, the formation of the aggregation state leads to blocked movement within the molecule, non-radiation attenuation channels are blocked and radiation attenuation is activated. At the same time, the highly distorted conformation hinders the π–π accumulation among molecules. Therefore, the luminous efficiency is improved and the compound emits strong fluorescence.

**Figure 2:**
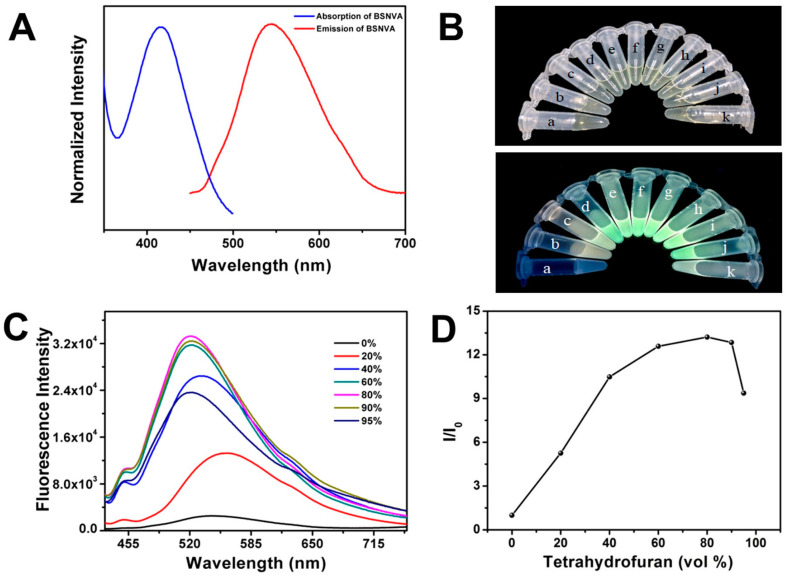
(**A**) Normalized absorption spectrum and FL spectrum of BSNVA in aqueous solution; (**B**) The photographs showed the visual color change (top) and fluorescent color change under 365 nm light (bottom) of the sensing system to different THF fractions (f_T_) at 0, 10, 20, 30, 40, 50, 60, 70, 80, 90 and 95%; (**C**) FL spectra and (**D**) corresponding FL Intensity ratio of BSNVA with different f_T_, where *I*_0_ is the FL intensity in water.

As shown in Figure 2A, the maximum absorption wavelength of BSNVA in aqueous solution was 410 nm. Its fluorescence was weak and its emission wavelength was 545 nm. Water was used as the good solvent and THF as the poor solvent to investigate the AIE performance of BSNVA. When the volume fraction (f_T_) of THF was 20%, BSNVA began to accumulate. With the increase in f_T_, the fluorescence gradually became stronger and reached the maximum when the f_T_ was 80%, which was 13 times higher than the fluorescence intensity in aqueous solution (Figure 2C,D). The corresponding solution photo is shown in Figure 2B. All the solutions were light yellow and transparent under sunlight. Under the ultraviolet light, the green fluorescence first increased and then decreased, which is consistent with the fluorescence spectra results.

### 3.3. Construction of MnO_2_–SiO_2_–AIE Nanocomposite System

**Figure 3:**
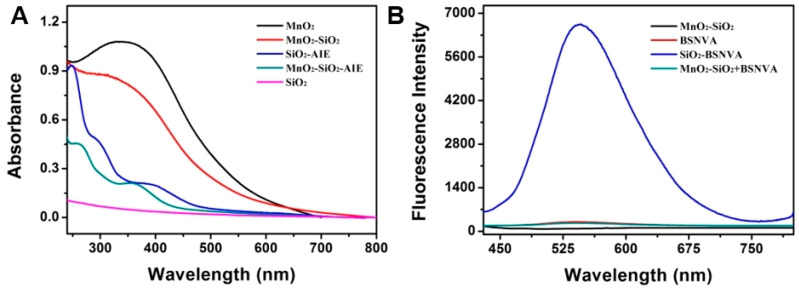
(**A**) UV–vis absorption spectra of MnO_2_ nanosheets (black line), MnO_2_–SiO_2_ NPs (red line), SiO_2_–BSNVA NPs (blue line), MnO_2_–SiO_2_ NPs–BSNVA (green line), SiO_2_–NH_2_ NSs (pink line), MnO_2_ + AA (yellow line). (**B**) Fluorescence emission spectra of MnO_2_–SiO_2_ NPs (black line), BSNVA (red line), MnO_2_–SiO_2_ NPs–BSNVA (green line), SiO_2_–BSNVA NPs (blue line).

By synthesizing the above nanoparticles and BSNVA molecules, the MnO_2_–SiO_2_–AIE nanocomposite system was constructed. As shown in Figure 3A, the prepared SiO_2_–NH_2_ NSs had no obvious UV–vis absorption in the range of 240–800 nm. The MnO_2_ nanosheet showed obvious optical properties and had an obvious UV–vis absorption at 370 nm. The MnO_2_–SiO_2_ complex also produced a UV–vis absorption at 370 nm, indicating that the MnO_2_ nanosheet had successfully adsorbed on the surface of SiO_2_–NH_2_ NSs. BSNVA has a negative charge and can be bounded to the surface of SiO_2_–NH_2_ NSs through electrostatic interaction. The SiO_2_–BSNVA complex produced an obvious UV–vis absorption peak at 410 nm, which is consistent with the UV–vis absorption of BSNVA, indicating that BSNVA had been successfully concentrated on the surface of SiO_2_–NH_2_ NSs. There was a blueshift in the UV–vis absorption of MnO_2_–SiO_2_–BSNVA complex at 410 nm, caused by the combination with MnO_2_ nanosheet. The results showed that the MnO_2_–SiO_2_ complex and SiO_2_–AIE complex had been successfully prepared.

In addition, the formation of nanocomposites was further confirmed via fluorescence measurements. The MnO_2_–SiO_2_ complex had no emission in aqueous solution (Figure 3B). BSNVA was soluble in water and hardly gave off light in aqueous solution. When BSNVA was combined with SiO_2_–NH_2_ NSs, the system emitted strong fluorescence. This was due to BSNVA molecules’ aggregation on the surface of SiO_2_–NH_2_ NSs, which limited their intramolecular motion, and, thus, turned on the fluorescence emission of BSNVA. It is worth noting that, when BSNVA was mixed with the MnO_2_–SiO_2_–NH_2_ nanosystem, its fluorescence remained almost unchanged. This was because the MnO_2_ nanosheet could wrap SiO_2_–NH_2_ NSs and shield the positive charge on the SiO_2_–NH_2_ NSs, resulting in the non-polymerization of BSNVA.

### 3.4. Concentration Optimization of MnO_2_ Nanosheet

**Figure 4:**
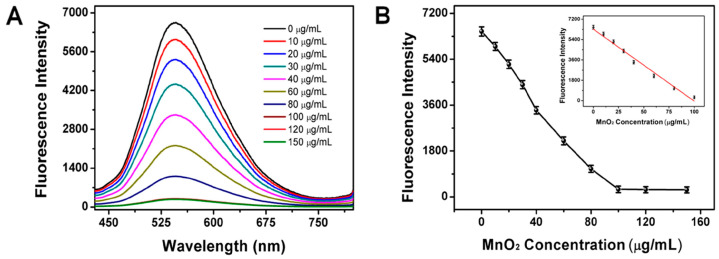
(**A**) Fluorescence emission spectra of SiO_2_–BSNVA NPs in the presence of varying concentrations of MnO_2_ nanosheets. (**B**) The corresponding fluorescence intensity change of SiO_2_–BSNVA NPs at 546 nm with various concentrations of MnO_2_ nanosheets.

The coating of the MnO_2_ nanosheet can shield the positive charge on the surface of SiO_2_–NH_2_ NSs, thus reducing the aggregation of BSNVA. In addition, it is also the target of reducing species and the key to the construction of an ALP sensor. Therefore, the concentration of MnO_2_ nanosheet was the main factor affecting the fluorescence intensity of the composite nanosystem. The fluorescence intensity of the system gradually decreased as the content of MnO_2_ nanosheet in the system gradually increased (Figure 4A,B). When the concentration of MnO_2_ nanosheet reached 100 μg/mL, the fluorescence intensity of the system hit its bottom, indicating that the SiO_2_–NH_2_ NSs were completely coated by MnO_2_ nanosheets. After that, the MnO_2_ nanosheets adsorbed on the surface of SiO_2_–NH_2_ NSs reached saturation and the fluorescence intensity of the system tended to be stable. As a result, 100 μg/mL was selected as the optimal concentration of MnO_2_ nanosheet for further experiments.

### 3.5. Detection of ALP by MnO_2_–SiO_2_–BSNVA Composite System

ALP can catalyze the dephosphorylation of AA2P to produce AA [38]. Based on the above principles, the applicability of the constructed composite nanosystem in ALP detection was further explored. As shown in Figure S5A, the specific absorbing peak of MnO_2_ nanosheet disappeared after adding AA, indicating that the MnO_2_ nanosheet can be decomposed by AA, which was consistent with TEM results. Fluorescence detection results showed that adding AA to the composite system could enhance the fluorescence of the composite nanosystem. The fluorescence spectra in Figure S5B shows that the fluorescence of the composite nanosystem was enhanced with AA being added to the MnO_2_–SiO_2_–BSNVA composite system. The reason is that AA induced the MnO_2_ nanosheet to decompose into Mn^2+^, which destroyed the composite structure and released SiO_2_ nanospheres. The positive charge on its surface was exposed, which facilitated the formation of the SiO_2_–BSNVA complex by combining with BSNVA in the system, resulting in BSNVA fluorescence enhancement. This result further proves the successful construction of nanocomplexes and also proves that the label-free and “turn-on” strategy proposed by us is feasible for ALP detection.

**Figure 5:**
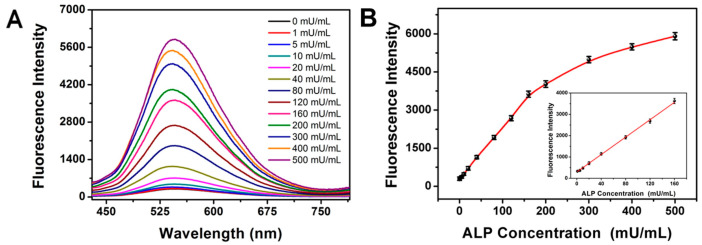
(**A**) Fluorescence emission spectra of the composite system in the presence of different concentrations of ALP. (**B**) The trend of fluorescence intensity in the composite system in the presence of different concentrations of ALP; the inset is the linear range of the fluorescence response of the composite system to ALP.

On the basis of the above studies, AA2P and ALP of different concentrations were added to the MnO_2_–SiO_2_–BSNVA composite system, so as to detect the fluorescence of the composite system under optimal experimental conditions. As shown in Figure 5A, the fluorescence intensity of the composite system was positively correlated with the concentration of ALP in the system. With the increase in ALP concentration, the fluorescence of the system recovered rapidly, almost reaching full recovery at 500 mU/mL. As shown in Figure 5B, the probe showed a good linear response to ALP in the range of 1–160 mU/mL; the linear equation was y = 297.2 + 20.41x (R^2^ = 0.998) and the detection limit was 0.38 mU/mL. The detection limit of this method is lower than those of other methods reported recently (Table 1).

**Figure 6:**
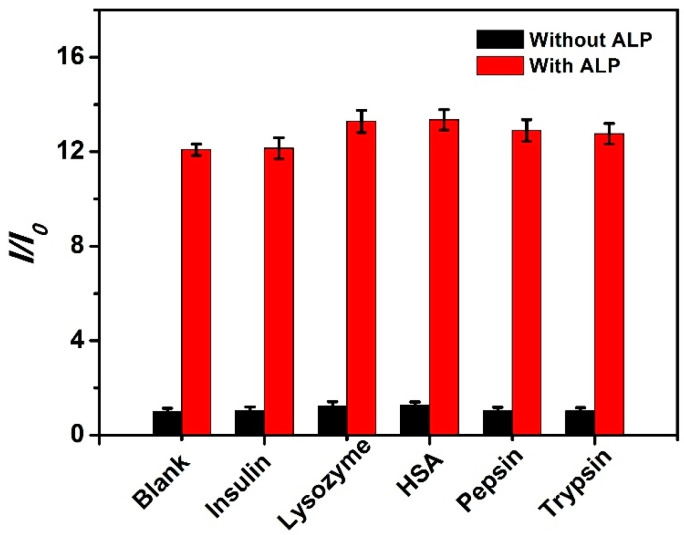
Changes in fluorescence intensity of MnO_2_–SiO_2_–BSNVA solution with addition of various substances (insulin, lysozyme, HSA, pepsin, trypsin). Black bars display the addition of foreign substances alone. Red bars display subsequent addition of ALP.

In addition, the selectivity of the MnO_2_–SiO_2_–BSNVA composite-sensing system for ALP detection was explored by comparing the fluorescence signals in the presence of other interfering substances (Figure 6). As demonstrated, the fluorescence of the sensing system remained unchanged or slightly changed upon addition of control proteins/enzymes (300 mU/mL), including insulin, lysozyme, human serum albumin, pepsin and trypsin. However, the fluorescence of the system could be enhanced when both the ALP (160 mU/mL) and the interfering substance were present, indicating that the system constructed has excellent selectivity for ALP.

### 3.6. Detection of ALP in Human Serum Sample

In order to evaluate the feasibility of the composite nanoprobe for practical application, the assay for detecting ALP in diluted human serum samples was carried out. To eliminate the interference of glutathione and cysteine, reduced N-ethylmaleimide was added to the serum samples [48]. As shown in Table 2, the average recoveries of the three real samples were 110.9, 108.2 and 107.0. The relative standard deviation (RSD) was between 2.24 and 2.81%, indicating that this composite nanoprobe could be used for detecting ALP in biological samples.

## 4. Conclusions

In conclusion, a “turn-on” composite nanosystem with high specificity and sensitivity based on AIE molecules was successfully constructed and used in the detection of ALP. Water-soluble BSNVA molecules, with good optical properties and negative charges in aqueous solution, were regarded as signal units. A MnO_2_ nanosheet could not only be used as a positive protectant to shield the positive charge on the surface of SiO_2_–NH_2_ NSs, but also as a response unit of the ALP-induced substrate decomposition reaction. It was decomposed to release SiO_2_–NH_2_ NSs, so that BSNVA molecules could accumulate on the surface of SiO_2_–NH_2_ NSs for fluorescence enhancement. The nanosensor did not require a complex modification process and overcame the disadvantages of traditional organic probes, such as poor water solubility and easy quenching at high concentration. In addition, the fluorescent probe was based on the “turn-on” strategy, which has the advantages of simple construction, fast response, low cost and being label-free. Furthermore, the combination of AIE molecules and inorganic nanomaterials provides a new perspective for the detection of enzyme activity, which has the potential to be applied in the diagnosis of diseases and biomedical research.

## Figures and Tables

**Table 1:** Comparison of the different methods for ALP detection.

Method	Detection Limit	Reference
Colorimetry	5.4 mU/mL	[39]
Colorimetry Colorimetry	0.87 mU/mL 0.7 mU/mL	[40] [41]
Electrochemical	3 mU/mL	[42]
Electrochemical	2.12 mU/mL	[12]
Fluorescence	0.87 mU/mL	[43]
Fluorescence Fluorescence Fluorescence Fluorescence	0.73 mU/mL 1.28 mU/mL 6.6 mU/mL 3.2 mU/mL	[44] [45] [46] [47]
Fluorescence	0.38 mU/mL	This work

**Table 2:** Detection of ALP in diluted serum samples.

Sample No.	Added	Detected	Recovery (%)	RSD (*n* = 5, %)
1	40 mU/mL	44.34 mU/mL	110.9	2.81
2	80 mU/mL	86.54 mU/mL	108.2	2.24
3	120 mU/mL	128.38 mU/mL	107.0	2.42

## Data Availability

The data supporting the findings of this study are available from the authors upon reasonable and appropriate request.

## Supplementary Materials

The following supporting information can be downloaded at: https://www.mdpi.com/article/10.3390/nano13142138/s1, Figure S1: The synthesis route of BSNVA; Figure S2: Zeta potential values of SiO_2_ NSs, SiO_2_–NH_2_ NSs, MnO_2_ nanosheet and MnO_2_–SiO_2_ composite. Figure S3: ^1^H NMR of BSNVA; Figure S4: The optimized molecular structure of BSNVA, which is represented as ball and stick, with carbon, oxygen, sulphur and hydrogen atoms colored cyan, red, yellow and gray, respectively; Figure S5: (A) UV–vis absorption spectra of MnO_2_ nanosheets (black line), MnO_2_ + AA (red line). (B) Fluorescence emission spectra of MnO_2_–SiO_2_ NPs + BSNVA (black line), MnO_2_–SiO_2_ NPs + BSNVA in the presence of AA (red line).
