# Supplementary material for: Construction of Aggregation-Induced Emission Molecule–MnO2 Composite Nanoprobe and Its Application in Alkaline Phosphatase Detection
Source: Nanomaterials (Basel). 2023 Jul 23;13(14):2138. doi: 10.3390/nano13142138 (PMC10383434; doi:10.3390/nano13142138)
Supplement: Supplementary file 1 [file nanomaterials-13-02138-s001.zip › nanomaterials-2490172-supplementary.pdf]

nanomaterials

Supplementary Materials

Construction of Aggregation-Induced Emission Molecule–MnO_2 Composite Nanoprobe and Its Application in Alkaline Phosphatase Detection

Yanyun Cui *, Jun Zhao and Huidan Li

College of Chemistry and Materials Engineering, Beijing Technology and Business University, Beijing 100048, China; zhaojun@st.btbu.edu.cn (J.Z.); lihuidan@st.btbu.edu.cn (H.L.)
* Correspondence: cuiyanyunns@126.com

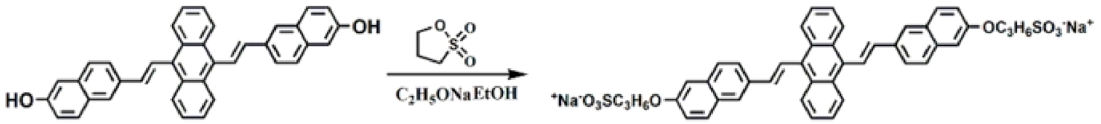

Figure S1. The synthesis route of BSNVA.

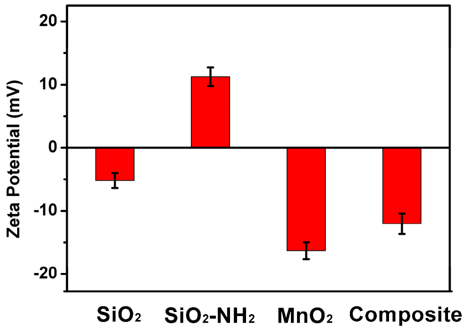

Figure S2. Zeta-potential values of SiO_2 NSs, SiO_2-NH_2 NSs, MnO_2 nanosheet and MnO_2-SiO_2 composite, and the corresponding potential values were respectively -5.21 eV, 11.27 eV, -16.32 eV and -12.03 eV.

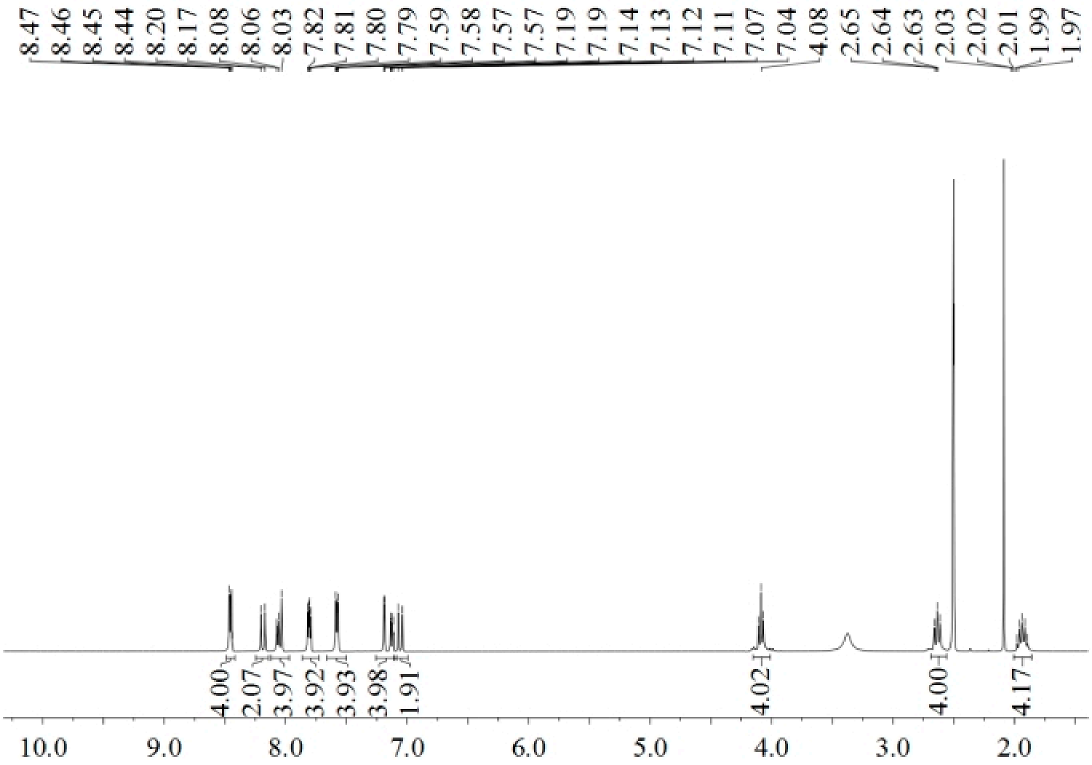

Figure S3. ^{1}H NMR of BSNVA.

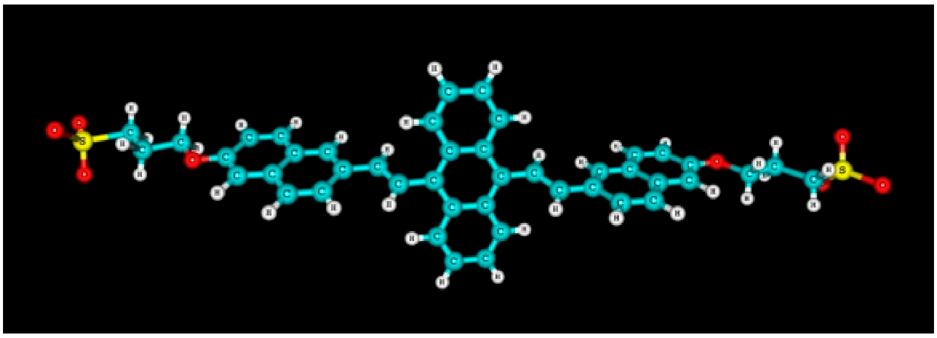

Figure S4. The optimized molecular structure of BSNVA, which is represented as ball and stick, with carbon, oxygen, sulphur, and hydrogen atoms colored cyan, red, yellow, and gray, respectively.

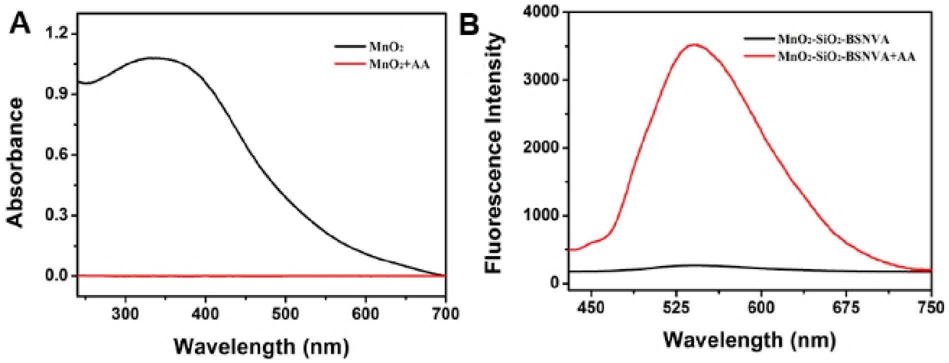

Figure S5. (A) UV-vis absorption spectra of MnO_2 nanosheets (black line), MnO_2+AA (red line). (B) Fluorescence emission spectra of MnO_2-SiO_2 NPs+BSNVA (black line), MnO_2-SiO_2 NPs+BSNVA in the present of AA (red line).
